# Genetic Variants at Chromosomes 2q35, 5p12, 6q25.1, 10q26.13, and 16q12.1 Influence the Risk of Breast Cancer in Men

**DOI:** 10.1371/journal.pgen.1002290

**Published:** 2011-09-15

**Authors:** Nick Orr, Rosie Cooke, Michael Jones, Olivia Fletcher, Frank Dudbridge, Sarah Chilcott-Burns, Katarzyna Tomczyk, Peter Broderick, Richard Houlston, Alan Ashworth, Anthony Swerdlow

**Affiliations:** 1 The Breakthrough Breast Cancer Research Centre, The Institute of Cancer Research, London, United Kingdom; 2 Section of Epidemiology, The Institute of Cancer Research, Sutton, United Kingdom; 3 Department of Non-Communicable Disease Epidemiology, The London School of Hygiene and Tropical Medicine, London, United Kingdom; 4 Section of Cancer Genetics, The Institute of Cancer Research, Sutton, United Kingdom; Stanford University School of Medicine, United States of America

## Abstract

Male breast cancer accounts for approximately 1% of all breast cancer. To date, risk factors for male breast cancer are poorly defined, but certain risk factors and genetic features appear common to both male and female breast cancer. Genome-wide association studies (GWAS) have recently identified common single nucleotide polymorphisms (SNPs) that influence female breast cancer risk; 12 of these have been independently replicated. To examine if these variants contribute to male breast cancer risk, we genotyped 433 male breast cancer cases and 1,569 controls. Five SNPs showed a statistically significant association with male breast cancer: rs13387042 (2q35) (odds ratio (OR)  = 1.30, p = 7.98×10^−4^), rs10941679 (5p12) (OR = 1.26, p = 0.007), rs9383938 (6q25.1) (OR = 1.39, p = 0.004), rs2981579 (*FGFR2*) (OR = 1.18, p = 0.03), and rs3803662 (*TOX3*) (OR = 1.48, p = 4.04×10^−6^). Comparing the ORs for male breast cancer with the published ORs for female breast cancer, three SNPs—rs13387042 (2q35), rs3803662 (*TOX3*), and rs6504950 (*COX11*)—showed significant differences in ORs (p<0.05) between sexes. Breast cancer is a heterogeneous disease; the relative risks associated with loci identified to date show subtype and, based on these data, gender specificity. Additional studies of well-defined patient subgroups could provide further insight into the biological basis of breast cancer development.

## Author Summary

Breast cancer is the most common female cancer in the United Kingdom but also occurs in men, albeit at a much lower frequency. Relatively little is known regarding risk factors for male breast cancer. Here, we examine the effect of common genetic variants that are known to be associated with female breast cancer to determine whether they also affect risk of male breast cancer. We show that certain of these variants are also associated with male breast cancer risk but that the magnitudes of their effects differ in males from females. Future analyses of the genetics of male breast cancer may shed light on the biology of both male and female breast cancer.

## Introduction

Breast cancer does not exclusively affect females. Around 300 men in the UK and 1,900 men in the US are diagnosed with the disease each year [1]. The average age at incidence of male breast cancer is somewhat different to that seen for female breast cancer, with the disease typically affecting men 5–10 years later than women. Perhaps because male breast cancer is not common, few risk factors have been demonstrated to influence disease risk, but tentative associations with obesity, lack of exercise, excess alcohol consumption, gynaecomastia, past benign breast disease, past liver disease, infertility, diabetes and exposure to ionising radiation have been suggested [2], [3].

Investigation of susceptibility genes for male breast cancer has been limited. It has however been shown that approximately 10% of men with breast cancer carry *BRCA2* mutations, while mutations in *BRCA1* are exceedingly rare [4]. The relative risk of breast cancer in men associated with *BRCA2* mutations is high [5]. Recently the *CHEK2* 1100delC variant has been found to give a 10-fold risk of male breast cancer independent of *BRCA1* or *BRCA2* [6]. Mutations in these genes are rare in the general population and it is likely that much of the genetic contribution to female breast cancer risk can be attributed to the co-inheritance of multiple low risk common variants [7]. Recent genome-wide association studies (GWAS) have shown associations between single nucleotide polymorphisms (SNPs) mapping to a dozen or more loci and female breast cancer risk in European populations, each conferring odds ratios (ORs) of 1.04–1.43 [8]–[14]. To explore the possibility that the same risk variants influence male breast cancer risk, we conducted a case-control study of male breast cancer, genotyping 12 SNPs annotating the loci that have the strongest and most consistent associations with female breast cancer.

## Materials and Methods

457 cases of male breast cancer were recruited in a population-based case-control study of the genetic, environmental and behavioral causes of male breast cancer being conducted in England and Wales. Potential cases were all men resident in these countries aged 18–79 with newly diagnosed breast cancer since January 1^st^, 2005, identified through notifications by treatment centres and systematic regular listings of cases from regional cancer registries. 98% of cases for whom registry data has been received have been histologically confirmed. The median age at diagnosis of cases was 65.5 years (interquartile range: 59–72).

A total of 1608 unmatched controls were available for genotyping; 535 men were ascertained through our ongoing breast cancer studies and a further 1073 were healthy male and female individuals from the UK Genetic Lung Cancer Predisposition Study (GELCAPS) [15]. The decision to include a second control set was made *a priori*, with the aim of increasing statistical power. We saw no evidence for an effect of control group on the overall effect estimate for each SNP. Collection of blood samples from all subjects was undertaken with informed consent and relevant ethical review committee approval.

DNA was extracted from venous blood samples using conventional methodologies and quantified by Picogreen (Invitrogen, Carlsbad CA). SNPs were chosen for analysis on the basis of validated associations with female breast cancer from recent GWAS [8]–[14]. Genotyping of rs11249433, rs13387042, rs4973768, rs10941679, rs16886165, rs9383938, rs13281615, rs865686, rs2981579, rs3817198, rs3803662 and rs6504950 was performed by allele-specific PCR using KASPar chemistry (Kbioscience, Hertfordshire, UK). Each DNA plate contained 5% sample duplication to assess genotyping concordance between duplicate pairs. We attempted to genotype 2119 samples (including duplicates, n = 54) and excluded samples (n = 49; 11 cases, 34 controls and four members of a duplicate pair) in which no-calls were observed for two or more SNPs. Genotyping QC statistics were therefore computed on 2070 samples (Figure S1). Final locus and sample completion rates were >99.9%. The mean genotype concordance between duplicate pairs was 99.8%. We excluded a further 18 subjects due to self-reported non-European ancestry (13 cases and 5 controls). No SNP genotypes showed significant deviation from the proportions expected under Hardy-Weinberg equilibrium in controls (Table S1).

ORs and 95% confidence intervals (CI) were calculated using unconditional logistic regression. The odds ratio for each SNP was determined by fitting multiplicative and unconstrained genetic models. P-values were computed from likelihood ratio test statistics. Case-only unconditional logistic regression was used to test the significance of association with age at diagnosis. Deviation of genotype proportions from Hardy-Weinberg equilibrium was assessed in controls using an exact test [16]. To compare formally the ORs in males with the equivalent published ORs for female disease, we assumed both sets of ORs were log-normally distributed. Then under the null hypothesis that the OR in males is equal to the OR in females, the difference between the estimated log ORs is normally distributed with mean zero and variance equal to the sum of the squared standard errors of the two estimates. From this we obtained a **χ^2^** statistic for each comparison (1 degree of freedom [d.f.]) and from the sum of the **χ^2^** statistics a global test for all comparisons (12 d.f.). Statistical analyses were performed using the Genotype Libraries and Utilities (GLU) package (http://code.google.com/p/glu-genetics) and R [17].

## Results/Discussion

433 male breast cancer cases and 1569 controls were successfully genotyped according to our predefined QC criteria. The majority of cases were diagnosed with invasive breast cancer (n = 399 (92%)) while a further 31 (7%) were ductal carcinoma in situ. Three cases (<1%) were of unknown histology. Table 1 shows the OR for male breast cancer associated with each of the 12 SNPs previously reported to be associated with female breast cancer risk. For five SNPs, rs13387042 (2q35), rs10941679 (5p12), rs9383938 (6q25.1), rs2981579 (*FGFR2*) and rs3803662 (*TOX3*), the risk allele for female breast cancer was associated with increased risk of male breast cancer (p<0.05). Two SNPs, rs13387042 (2q35) and rs3803662 (*TOX3*), remained significant below the Bonferroni adjusted threshold for independent tests of p<4.12×10^−3^.

**Table 1:** Risk estimates for male breast cancer conferred by 12 loci identified through GWAS of female breast cancer.

SNP	Chromosome	Gene	Risk Allele^a^	Risk Allele Freq	Male breast cancer				Female breast cancer^b^	
					OR_(het)_	OR_(hom)_	OR_(trend)_	P_(trend)_	OR_(trend)_	P_(trend)_
rs11249433	1p11.2	*NOTCH2*	C	0.42	1.07	1.28	1.12	0.13	1.20 [^13^]	4.48−10^−13^
					(0.84–1.36)	(0.94–1.73)	(0.97–1.31)		(1.14–1.25)	
rs13387042	2q35		A	0.50	1.31	1.69	1.30	7.98−10^−04^	1.12 [^11^]	1.00−10^−19^
					(1.00–1.73)	(1.24–2.29)	(1.11–1.51)		(1.09–1.15)	
rs4973768	3p24.1	*SLC4A7*	T	0.47	1.05	1.28	1.13	0.11	1.11 [^8^]	1.40−10^−18^
					(0.81–1.36)	(0.95–1.74)	(0.97–1.32)		(1.08–1.13)	
rs10941679	5p12		G	0.27	1.28	1.54	1.26	0.007	1.19 [^12^]	2.90−10^−11^
					(1.02–1.60)	(1.04–2.29)	(1.07–1.48)		(1.13–1.26)	
rs16886165	5q11.2	*MAP3K1*	G	0.16	0.99	0.85	0.97	0.77	1.23 [^13^]	5.00−10^−07^
					(0.78–1.26)	(0.41–1.78)	(0.79–1.19)		(1.12–1.35)	
rs9383938	6q25.1	*ESR1*	T	0.09	1.31	2.79	1.39	0.01	1.18 [^10^]	1.41−10^−07^
					(0.99–1.73)	(1.11–7.00)	(1.09–1.78)		(1.11–1.26)	
rs13281615	8q24.21		G	0.41	0.97	1.20	1.07	0.37	1.08 [^9^]	5.00−10^−12^
					(0.76–1.23)	(0.88–1.64)	(0.92–1.25)		(1.05–1.11)	
rs865686	9q31.2		T	0.62	1.07	1.10	1.04	0.62	1.11[^10^]	1.75−10^−10^
					(0.77–1.49)	(0.78–1.53)	(0.89–1.22)		(1.04 -1.19)	
rs2981579	10q26.13	*FGFR2*	T	0.42	1.06	1.43	1.18	0.03	1.26 [^9]^, [14]	2.00−10^−76^
					(0.83–1.36)	(1.06–1.94)	(1.02–1.38)		(1.23–1.30)	
rs3817198	11p15.5	*LSP1*	C	0.32	0.94	0.87	0.93	0.39	1.07 [^9^]	3.00−10^−09^
					(0.75–1.89)	(0.60–1.25)	(0.79–1.09)		(1.04 -1.11)	
rs3803662	16q12.1	*TOX3*	T	0.27	1.48	2.21	1.48	4.04−10^−06^	1.20 [^9^]	1.00−10^−36^
					(1.18–1.85)	(1.50–3.25)	(1.26–1.75)		(1.16–1.24)	
rs6504950	17q22	*COX11*	G	0.72	0.86	0.79	0.90	0.22	1.05 [^8^]	1.40×10^−08^
					(0.58–1.28)	(0.53–1.17)	(0.76–1.06)		(1.03–1.07)	

a Risk allele for female breast cancer.

b Female association statistics and effect estimates from previously published data.

Comparing OR_male_ estimates with those for female breast cancer (OR_female_) there were two SNPs, rs13387042 (2q35) and rs3803882 (*TOX3*) for which the OR_male_ was significantly higher than the OR_female_, albeit not after adjusting for multiple testing (rs13387042, OR_male_:OR_female_ p = 0.03; rs3803882, OR_male_:OR_female_ p = 0.04; Table 2). rs3803662 (*TOX3*) showed the strongest association with male breast cancer (OR_male_  = 1.48; 95% CI 1.26–1.75, p = 4.04×10^−6^) with an excess relative risk that was more than twice the female estimate (OR_female_  = 1.20; 95% CI 1.16–1.24) [9]. Similarly, the excess risk conferred by rs13387042 (2q35) in males (OR_male_  = 1.30; 95% CI 1.11–1.51, p = 7.98×10^−4^) was more than double that observed in females (OR_female_  = 1.12; 95% CI 1.09–1.15) [11].

**Table 2:** Ratio of OR_male_:OR_female_ for 12 risk loci identified by genome-wide association studies of female breast cancer.

SNP	Chromosome	OR_male_:OR_female_ (95% CI)	χ^2^	P-value^a^
rs11249433	1p11.2	0.95 (0.80–1.11)	0.425	0.50
rs13387042	2q35	1.19 (1.01–1.39)	4.538	0.03
rs4973768	3p24.1	1.07 (0.91–1.25)	0.681	0.41
rs10941679	5p12	1.03 (0.86–1.22)	0.087	0.77
rs16886165	5q11.2	0.81 (0.65–1.02)	3.116	0.08
rs9383938	6q25.1	1.22 (0.95–1.58)	2.400	0.12
rs13281615	8q24.21	1.02 (0.87–1.20)	0.036	0.85
rs865686	9q31.2	0.95 (0.80–1.13)	0.293	0.59
rs2981579	10q26.13	0.96 (0.82–1.12)	0.315	0.57
rs3817198	11p15.5	0.87 (0.74–1.03)	2.646	0.10
rs3803662	16q12.1	1.19 (1.01–1.42)	4.118	0.04
rs6504950	17q22	0.84 (0.71–0.99)	4.086	0.04
**All SNPs combined**			**22.769**	**0.03**

a P value for null hypothesis of no difference between OR_male_ and OR_female_ for each SNP individually and for all SNPs combined (in bold).

For one SNP (rs6504950, *COX11*) the OR_male_ was in the opposite direction to that reported for female breast cancer (OR_male_  = 0.90; 95% CI 0.76–1.06, OR_female_  = 1.05; 95% CI 1.03–1.07) [8], [9] and was inconsistent with the female estimate (OR_male_:OR_female_ p = 0.04; Table 2). For the other nine SNPs that we tested the OR_male_ estimates were consistent with the OR_female_ estimates. Comparing the combined estimates of all 12 SNPs, however, there was nominal evidence that the male ORs differed from the female ORs (p = 0.03; Table 2).

The frequency of female breast tumors that are estrogen receptor (ER) positive varies, particularly according to menopausal status at diagnosis [18]. Based on a sample of almost 3,000 patients the proportion is typically between 64% and 79% [18]. In contrast, male breast tumors, tend to be overwhelmingly ER-positive (>90%) [19]. In the current study estrogen receptor status was known for 251 male breast cancer cases, 246 (98%) of which had ER-positive tumors. For nine of the 12 SNPs that we genotyped, OR_female_ estimates stratified according to ER status have been reported for Caucasian populations (Tables S2a and S2b). In females, the OR for ER-positive disease is stronger than the OR for ER-negative disease for all nine of these loci and this difference is significant for all but two of them (rs16886165 (*MAP3KI*) and rs3817198 (*LSP1*) [8], [11], [20]. Given the predominance of ER-positive tumors in male disease we also compared the OR_male_ with the OR_female_ for ER-positive disease (Table S2a). There was nominally significant evidence overall that the male ORs differed from those for ER-positive female disease (p = 0.05). We also tested for a difference between the OR_male_ estimates for these nine SNPs and the OR_female_ estimates for ER-negative disease (Table S2b); there was stronger evidence of a difference (p = 0.01).

Finally, we assessed the relationship between genotype and age at onset of male breast cancer (Table S3) for each of the 12 loci. There was no evidence for a trend with age at diagnosis.

We have shown, for the first time that common genetic variants influence susceptibility to male breast cancer. Furthermore we have demonstrated that for at least a subset of known susceptibility loci the risk allele for female breast cancer is also associated with increased risk of disease in males. To our knowledge these 433 male breast cancer cases represent the largest single series to date; despite this, we lacked power to detect modest relative risks for all but the most common variants. For example we had only 40% power to detect an OR of 1.15 for a variant with a minor allele frequency of 30% at a significance level of 5%. The lack of a statistically significant association with male breast cancer risk for seven of the 12 SNPs that we tested may, therefore, simply reflect a lack of power.

Notably, for two of the three SNPs for which the OR_male_ was inconsistent with the OR_female_ (rs13387042 (2q35) and rs3803882 (*TOX3*)) the association in males was stronger than that in females. While the OR_male_ estimates were slightly closer to the ORs for ER-positive disease in females, it is noticeable that these are the two SNPs that show the largest effects on ER-negative disease risk in females. Although the significance of this observation, if any, is not yet clear, our data on male breast cancer alongside the published associations with female breast cancer, clearly implicate the 2q35 and 16q12.1 loci in the aetiology of breast cancer, irrespective of gender and tumor pathology.

Given that the majority of female breast cancer risk loci identified to date demonstrate a degree of specificity for ER-positive or ER-negative disease [8], [11], [20], [21] it seems likely that subtype specific GWAS will lead to the identification of additional risk loci. Our analyses suggest that GWAS of male breast cancer may also lead to the identification of novel breast cancer risk loci in males and that these should provide further insight into the biological basis of male and female breast cancer development.

## Supporting Information

Figure S1 Sample exclusion schema.
(DOCX)

Table S1 P values for exact test of deviation from genotype proportions expected under Hardy-Weinberg equilibrium in controls.
(DOCX)

Table S2 Ratio of OR estimate for male breast cancer and OR estimate for a) estrogen receptor positive female breast cancer and for b) estrogen receptor negative female breast cancer for nine SNPs for which stratified estimates have been reported.
(DOCX)

Table S3 Odds ratios and 95% confidence intervals for each locus modeled multiplicatively in cases aged less than 60 years (n = 119), between 60 and 69 years (n = 153) and cases aged 70 years and greater (n = 161) versus all controls (n = 1569).
(DOCX)
