# Supplementary material for: Genetic Variants at Chromosomes 2q35, 5p12, 6q25.1, 10q26.13, and 16q12.1 Influence the Risk of Breast Cancer in Men
Source: PLoS Genet. 2011 Sep 15;7(9):e1002290. doi: 10.1371/journal.pgen.1002290 (PMC3174231; doi:10.1371/journal.pgen.1002290)
Supplement: Figure S1 — Sample exclusion schema. (DOCX) [file pgen.1002290.s001.docx]

**KASPar Genotyping**

**Cases: 457**

**Controls: 1608**

**Duplicates: 54**

**Total: 2119**

**Exclusions (Ethnicity)**

**Cases: 13**

**Controls: 5**

**Total: 18**

**GELCAPS**

**Controls: 1073**

**Male Breast Cancer Study**

**Cases: 457**

**Controls: 535**

**Removed the duplicates (n=50)**

**Final Dataset**

**Cases: 433**

**Controls: 1569**

**Total: 2002**

**Exclusions (Completion)**

**Cases: 11**

**Controls: 34**

**Duplicates: 4**

**Total: 49**

Figure S1
