# Supplementary material for: Genetic Variants at Chromosomes 2q35, 5p12, 6q25.1, 10q26.13, and 16q12.1 Influence the Risk of Breast Cancer in Men
Source: PLoS Genet. 2011 Sep 15;7(9):e1002290. doi: 10.1371/journal.pgen.1002290 (PMC3174231; doi:10.1371/journal.pgen.1002290)
Supplement: Table S1 — P values for exact test of deviation from genotype proportions expected under Hardy-Weinberg equilibrium in controls. (DOCX) [file pgen.1002290.s002.docx]

Table S1

| SNP | P-value  Male Breast Controls | P-value GELCAPS |
| --- | --- | --- |
| rs11249433 | 0.70 | 0.59 |
| rs13387042 | 0.42 | 0.79 |
| rs4973768 | 0.62 | 0.42 |
| rs10941679 | 1.00 | 0.49 |
| rs16886165 | 0.35 | 0.08 |
| rs9383938 | 0.32 | 0.35 |
| rs13281615 | 0.11 | 0.52 |
| rs865686 | 0.84 | 0.93 |
| rs2981579 | 0.85 | 1.00 |
| rs3817198 | 0.12 | 0.77 |
| rs3803662 | 0.81 | 0.31 |
| rs6504950 | 0.64 | 0.38 |
