# Supplementary material for: Genetic Variants at Chromosomes 2q35, 5p12, 6q25.1, 10q26.13, and 16q12.1 Influence the Risk of Breast Cancer in Men
Source: PLoS Genet. 2011 Sep 15;7(9):e1002290. doi: 10.1371/journal.pgen.1002290 (PMC3174231; doi:10.1371/journal.pgen.1002290)
Supplement: Table S2 — Ratio of OR estimate for male breast cancer and OR estimate for a) estrogen receptor positive female breast cancer and for b) estrogen receptor negative female breast cancer for nine SNPs for which stratified estimates have been reported. (DOCX) [file pgen.1002290.s003.docx]

Table S2a

| **SNP** | **Chromosome** | **OR_male_:OR_female_ (95% CI)** | **χ^2^** | **P-value^a^** |
| --- | --- | --- | --- | --- |
| rs13387042 | 2q35 | 1.17 (0.99 - 1.37) | 3.569 | 0.06 |
| rs4973768 | 3p24.1 | 1.06 (0.91 - 1.24) | 0.509 | 0.48 |
| rs10941679 | 5p12 | 0.96 (0.80 - 1.15) | 0.175 | 0.68 |
| rs16886165 | 5q11.2 | 0.89 (0.72 - 1.10) | 1.102 | 0.29 |
| rs13281615 | 8q24.21 | 0.97 (0.83 - 1.14) | 0.131 | 0.72 |
| rs2981579 | 10q26.13 | 0.92 (0.78 - 1.07) | 1.086 | 0.30 |
| rs3817198 | 11p15.5 | 0.87 (0.74 - 1.03) | 2.646 | 0.10 |
| rs3803662 | 16q12.1 | 1.17 (0.98 - 1.38) | 3.061 | 0.08 |
| rs6504950 | 17q22 | 0.83 (0.70 - 0.99) | 4.471 | 0.03 |
| **All SNPs combined** | |  | **16.75** | **0.05** |

^a^P value for null hypothesis of no difference between OR_male_ and OR_female_ for each

SNP individually and for all SNPs combined (in bold).

Stratified results have been reported for rs2046210 [22] annotating 6q25.1 in a study of Asian but not Caucasian breast cancer cases and controls

Table S2b

| **SNP** | **Chromosome** | **OR_male_:OR_female_ (95% CI)** | **χ^2^** | **P-value^a^** |
| --- | --- | --- | --- | --- |
| rs13387042 | 2q35 | 1.21 (1.02 - 1.42) | 5.053 | 0.02 |
| rs4973768 | 3p24.1 | 1.12 (0.95 - 1.32) | 1.840 | 0.17 |
| rs10941679 | 5p12 | 1.16 (0.94 - 1.44) | 1.932 | 0.16 |
| rs16886165 | 5q11.2 | 0.93 (0.75 - 1.16) | 0.372 | 0.54 |
| rs13281615 | 8q24.21 | 1.07 (0.90 - 1.27) | 0.511 | 0.47 |
| rs2981579 | 10q26.13 | 1.12 (0.95 - 1.31) | 1.767 | 0.18 |
| rs3817198 | 11p15.5 | 0.90 (0.76 - 1.06) | 1.570 | 0.21 |
| rs3803662 | 16q12.1 | 1.26 (1.06 - 1.50) | 6.639 | 0.01 |
| rs6504950 | 17q22 | 0.91 (0.76 - 1.08) | 1.119 | 0.29 |
| **All SNPs combined** | |  | **20.804** | **0.01** |

^a^P value for null hypothesis of no difference between OR_male_ and OR_female_ for each

SNP individually and for all SNPs combined (in bold).

Stratified results have been reported for rs2046210 [22] annotating 6q25.1 in a study of Asian but not Caucasian breast cancer cases and controls
