# Supplementary material for: Genetic Variants at Chromosomes 2q35, 5p12, 6q25.1, 10q26.13, and 16q12.1 Influence the Risk of Breast Cancer in Men
Source: PLoS Genet. 2011 Sep 15;7(9):e1002290. doi: 10.1371/journal.pgen.1002290 (PMC3174231; doi:10.1371/journal.pgen.1002290)
Supplement: Table S3 — Odds ratios and 95% confidence intervals for each locus modeled multiplicatively in cases aged less than 60 years (n = 119), between 60 and 69 years (n = 153) and cases aged 70 years and greater (n = 161) versus all controls (n = 1569). (DOCX) [file pgen.1002290.s004.docx]

Table S3

| **SNP** | **Age <60** | **Age 60-69** | **Age 70+** | **P-value^a^** |
| --- | --- | --- | --- | --- |
| rs11249433 | 1.04 (0.80-1.36) | 1.26 (1.00-1.60) | 1.06 (0.84-1.34) | 1.00 |
| rs13387042 | 1.43 (1.09-1.86) | 1.22 (0.96-1.55) | 1.28 (1.02-1.62) | 0.60 |
| rs4973768 | 1.25 (0.95-1.64) | 1.25 (0.98-1.59) | 0.96 (0.76-1.21) | 0.12 |
| rs10941679 | 1.32 (1.00-1.76) | 1.38 (1.07-1.78) | 1.10 (0.85-1.42) | 0.26 |
| rs16886165 | 0.88 (0.60-1.29) | 0.94 (0.67-1.30) | 1.07 (0.78-1.45) | 0.42 |
| rs9383938 | 1.09 (0.69-1.74) | 1.75 (1.23-2.48) | 1.30 (0.89-1.90) | 0.69 |
| rs13281615 | 1.13 (0.86-1.48) | 1.18 (0.92-1.50) | 0.95 (0.75-1.21) | 0.30 |
| rs865686 | 1.27 (0.96-1.69) | 0.99 (0.78-1.26) | 0.95 (0.75-1.20) | 0.11 |
| rs2981579 | 1.21 (0.93-1.59) | 0.99 (0.78-1.26) | 1.36 (1.08-1.72) | 0.40 |
| rs3817198 | 0.81 (0.61-1.09) | 1.19 (0.93-1.51) | 0.80 (0.62-1.03) | 0.75 |
| rs3803662 | 1.44 (1.08-1.93) | 1.63 (1.27-2.10) | 1.38 (1.07-1.78) | 0.74 |
| rs6504950 | 0.88 (0.66-1.17) | 1.08 (0.82-1.41) | 0.78 (0.61-1.00) | 0.42 |

^a^P-values from case-only likelihood ratio tests with one degree of freedom.
